# Dexmedetomidine Inhibition of GABA Neurons in the Ventral Tegmental Area Attenuates the Depth of Sedation in Mice

**DOI:** 10.1002/brb3.71835

**Published:** 2026-10-14

**Authors:** Jin Rao, Gaolin Qiu, Jiansheng Wang, Yuanhai Li

**Affiliations:** ^1^ Department of Anesthesiology The First Affiliated Hospital of Anhui Medical University Hefei Anhui Province China; ^2^ Department of Rehabilitation Medicine The First Affiliated Hospital of Anhui Medical University Hefei Anhui Province China

**Keywords:** arousable sedation, dexmedetomidine, neural mechanisms, ventral tegmental area

## Abstract

**Introduction:**

Dexmedetomidine is a sedative anesthetic with a unique ability to produce an arousable state of sedation. However, the neural mechanisms underlying this arousable sedation remain incompletely understood. Previous studies have implicated dopaminergic neurons in the ventral tegmental area (VTA) in mediating this effect, but the upstream neural circuit mechanisms remain unclear. Furthermore, although VTA GABAergic neurons exert robust inhibitory effects on dopaminergic neurons, their specific role in dexmedetomidine‐induced arousable sedation remains unclear.

**Methods:**

Vgat‐Cre transgenic mice were used to investigate the role of VTA GABAergic neurons in dexmedetomidine‐induced arousable sedation. Electroencephalography (EEG), fiber photometry, calcium imaging, GABA neurotransmitter sensing, chemogenetic manipulation, and immunohistochemistry were used to assess neuronal activity, GABAergic signaling, and behavioral responses to dexmedetomidine.

**Results:**

Fiber photometry using the calcium indicator GCaMP6m revealed that dexmedetomidine inhibited VTA GABAergic neurons. Fiber photometry with the GABA neurotransmitter sensor iGABASnFR demonstrated that dexmedetomidine increased GABAergic input to VTA GABAergic neurons. Chemogenetic manipulation combined with EEG demonstrated that activating VTA GABAergic neurons deepened dexmedetomidine‐induced sedation, whereas inhibiting these neurons attenuated sedation.

**Conclusion:**

These findings suggest that dexmedetomidine increases GABAergic input to VTA GABAergic neurons and suppresses their activity, potentially contributing to its arousable sedative effects. Further studies are needed to establish the causal role of this neural circuit mechanism.

## Graphical Abstract

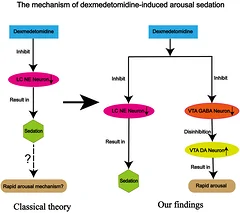

## 1 Introduction

Dexmedetomidine is a widely used clinical sedative and a highly selective α2‐adrenergic receptor agonist (Mantz et al. 2011; Walsh et al. 2025). Unlike many other sedatives, its sedative effect is characterized by a readily arousable state (Qiu et al. 2026; Qiu et al. 2020; Sleigh et al. 2018). Accordingly, dexmedetomidine is widely used in functional neurosurgery (Lin et al. 2019; Rozet 2008; Tasbihgou et al. 2021) and intensive care units (ICUs) (Heybati et al. 2022; Su et al. 2016), where sedation that permits patient arousal is desirable. However, the neural mechanisms underlying this arousable sedation remain incompletely understood. The brainstem locus coeruleus (LC) has long been considered a key region mediating dexmedetomidine‐induced sedation (Anuncibay Soto et al. 2025; Nelson et al. 2003). Local administration of dexmedetomidine into the LC induces sedation in rats, supporting a direct role for this region (Nelson et al. 2003). As the primary source of noradrenergic neurons in the central nervous system, the LC expresses α2‐adrenergic receptors on norepinephrine (NE) neurons. Dexmedetomidine activates these receptors and inhibits LC neuronal activity through G‐protein‐coupled mechanisms (Qiu et al. 2024), thereby reducing NE release throughout the brain. Because the LC‑NE system is a major arousal‑promoting system, its inhibition by dexmedetomidine provides a plausible mechanism for sedation. However, LC‐NE inhibition alone does not fully explain the characteristic readily arousable state produced by dexmedetomidine. Our previous studies demonstrated that dexmedetomidine activates dopamine (DA) neurons in the ventral tegmental area (VTA), resulting in increased DA neurotransmitter release in downstream regions, including the medial prefrontal cortex and nucleus accumbens (Qiu et al. 2020). The VTA DA system is also an important regulator of arousal (Taylor et al. 2016; Vincent et al. 2024). In vivo activation of VTA DA neurons promotes wakefulness in mice, whereas inhibition of this system facilitates sleep (Eban‐Rothschild et al. 2016; Taylor et al. 2016). Consistent with these findings, activating VTA DA neurons attenuates dexmedetomidine‐induced sedation, whereas inhibiting these neurons potentiates its sedative effect (Qiu et al. 2020). Thus, in addition to inhibiting the LC‑NE system, dexmedetomidine may modulate the VTA DA system, suggesting that the readily arousable nature of its sedation may arise from coordinated effects on multiple neural circuits involved in sedation and arousal. The interaction between these two opposing systems may contribute to its distinctive sedative state induced by dexmedetomidine. Nevertheless, given the intricate organization of the central nervous system, other neuronal populations may also contribute to this arousable phenotype. VTA GABAergic neurons are important regulators of sleep‐wake states (Lee et al. 2001; Oishi et al. 2023; Yu et al. 2019) and anesthesia (Li et al. 2025; Yin et al. 2019). Previous studies have shown that activation of VTA GABAergic neurons deepens isoflurane anesthesia (Wang et al. 2025; Yin et al. 2019). Similarly, activation of these neurons promotes sleep in mice, whereas their inhibition promotes wakefulness (Chowdhury et al. 2019). Importantly, VTA GABAergic neurons exert strong inhibitory control over VTA DA neurons (Tao et al. 2023; Tao et al. 2024; Yu et al. 2019). However, whether VTA GABAergic neurons are involved in mediating the readily arousable sedation induced by dexmedetomidine remains unknown. Therefore, this study employed electroencephalography (EEG), chemogenetics, fiber photometry, neurotransmitter sensors, and calcium indicators to investigate the role of VTA GABAergic neurons in dexmedetomidine‑induced arousable sedation. By elucidating the underlying neural mechanisms, this study aims to provide a theoretical basis for identifying potential targets for the development of more precise sedative agents that permit easy arousal.

## 2 Methods

### 2.1 Animals

Our experiments were conducted in accordance with the Animal Research: Reporting of In Vivo Experiments 2.0 Essential 10 guidelines (Percie du Sert et al. 2020). All animal care and experimental procedures complied with institutional policies and governmental regulations and were approved by the Animal Ethics Committee of Anhui Medical University (Ethical Approval No. LLSC20210042). The following mouse strains were used: Vgat‐Cre mice (8–12 weeks old; Slc32a1^2(cre)Lowl^; Stock No. 028862; The Jackson Laboratory) and C57BL/6J mice (8–12 weeks old; GemPharmatech Co., Ltd., Nanjing, China). Mice were housed in groups (up to five per cage) under a 12‐h light/dark cycle (lights on from 8:00 a.m. to 8:00 p.m.), with food and water available ad libitum. The housing environment was maintained at 24°C ± 2°C and 55% ± 5% relative humidity. In all experiments, dexmedetomidine was administered at a dose of 40 µg/kg.

### 2.2 Virus Injection and Optical Fiber Implantation

The procedures were performed as described in our previous studies (Qiu et al. 2026; Zhu et al. 2023). Mice were initially anesthetized with 4% isoflurane and maintained under 1.5% isoflurane anesthesia, with 50% oxygen delivered intranasally at a flow rate of 0.8–1.2 L/min. Body temperature was maintained at 37°C using an electric heating pad (RWD Life Science, Shenzhen, China). Each mouse was secured in a stereotaxic frame (RWD Life Science) with the skull oriented parallel to the reference plane, and a small burr hole was carefully drilled. Using a microsyringe pump (RWD Life Science), 300 nL of viral vector was infused into the VTA over 5 min through a precision glass pipette (coordinates: –3.1 mm AP, ±0.45 mm ML, –4.25 mm DV). Following infusion, the pipette was left in place for at least 15 min to allow adequate diffusion. For fiber photometry experiments, the calcium indicator GCaMP6m was injected unilaterally, whereas bilateral injections were performed for chemogenetic studies. For optical fiber implantation, a fiber‐optic cannula (200 µm diameter, 0.39 numerical aperture [NA]; RWD Life Science) was positioned 10 µm above the injection site, with its depth precisely controlled using the stereotaxic manipulator. The implant was secured with Super‐Bond C&B dental acrylic (NISSIN, Yokohama, Japan). After the cement had fully cured, mice were removed from the stereotaxic apparatus and placed on a 37°C warming blanket for recovery before being returned to their home cages.

### 2.3 Selective AAV Expression in VTA GABAergic Neurons Using the Cre‐loxP System

To achieve selective AAV expression in VTA GABAergic neurons, we used the Cre‐loxP recombination system. Briefly, Vgat‐Cre mice, in which Cre recombinase is selectively expressed in GABAergic neurons, received a Cre‐dependent AAV vector injection into the VTA. The AAV vector contained the gene of interest in an inverted orientation between two loxP sites (double‐floxed inverted open reading frame [DIO]). In the absence of Cre recombinase, the inverted sequence is not expressed. In Cre‐positive GABAergic neurons, Cre‐mediated recombination inverts the floxed cassette into the correct orientation, enabling transcription and expression of the target gene. This approach enables cell‐type‐specific transgene expression in VTA GABAergic neurons while minimizing expression in non‐GABAergic cells.

### 2.4 In Vivo Fiber Photometry

Fiber photometry was performed according to the protocols established in our previous study (Qiu et al. 2020). Fluorescence signals were acquired using light‐emitting diodes (480 and 405 nm; Lumileds, Shanghai, China). The excitation beams were reflected by a dichroic mirror (Edmund Optics, Barrington, IL, USA) and focused through a 20× objective lens (NA 0.4; Olympus, Tokyo, Japan). To minimize photobleaching, the excitation light power was maintained at 40–70 µW. Fluorescence emissions were filtered using a bandpass filter (87753; Edmund Optics) and detected with a photomultiplier tube (H10721; Hamamatsu, Shizuoka, Japan). The photomultiplier tube output was converted to voltage using an amplifier and then passed through a low‐pass filter with a 35‐Hz cutoff (Thinkertech, Nanjing, China). Analog voltage signals were digitized at 100 Hz and recorded using fiber photometry software (Thinkertech). For behavioral recordings, mice were connected to the fiber photometry system via a head‐mounted patch cord, allowing unrestricted movement. Following each recording session, photometry data were exported as MATLAB (.mat) files. Fluorescence changes were expressed as Δ*F*/*F*, calculated as (*F* − *F*_0_)/*F*_0_, where *F*_0_ represents the mean baseline fluorescence during a 100‐s control period.

### 2.5 Electroencephalography

EEG recordings were performed according to the methodology described in our previous study (Qiu et al. 2026). Mice were initially anesthetized with 4% isoflurane and maintained at 1.5% isoflurane throughout the procedure. Following placement in a stereotaxic apparatus, body temperature was maintained at 37°C using an electric heating pad. Three precise burr holes were drilled in the skull using a carbide drill: one for a reference electrode over the left frontal cortex (1 mm anterior to bregma and 1.5 mm lateral to the midline), one over the right parietal cortex (1 mm anterior to lambda and 1.5 mm lateral to the midline), and one over the left parietal cortex (1 mm anterior to lambda and 1.5 mm lateral to the midline). EEG recording electrodes were inserted through the burr holes. Stainless steel screws with attached electrode wires were secured in the skull, and all electrodes were anchored with dental acrylic. After the acrylic had fully cured, mice were placed on a heating pad to recover from anesthesia before being returned to their home cages and holding facility. Behavioral experiments assessing dexmedetomidine‐induced sedation were conducted 14 days post‐surgery. EEG signals were digitized using PowerLab and LabChart systems (AD Instruments, Dunedin, New Zealand), and power spectral density was calculated using fast Fourier transform. Because dexmedetomidine sedation resulted in minimal motion artifacts, epochs containing overt artifacts were excluded by visual inspection, supplemented by an amplitude threshold of ±700 µV. Sedation states were evaluated based on changes in relative power across the following frequency bands: delta (δ, 0.5–4 Hz), theta (θ, 4–7 Hz), alpha (α, 8–15 Hz), and beta (β, 16–30 Hz) (Guo et al. 2021; Qiu et al. 2020).

### 2.6 Histology

After completion of all experimental procedures, immunostaining was performed to label dopamine neurons in the VTA. Mice were anesthetized by intraperitoneal injection of pentobarbital (100 mg/kg) and subsequently perfused intracardially with normal saline. After the blood had been largely cleared, the animals were perfused with 4% paraformaldehyde for tissue fixation. Perfusion was continued until the limbs became rigid. To ensure complete fixation, the heads were removed and immersed in 4% paraformaldehyde overnight at room temperature. The brains were extracted the following day and cryoprotected by immersion in phosphate‐buffered saline containing 30% sucrose for 24–48 h.

Coronal brain sections (40 µm thick) were prepared using a freezing microtome (CM3050S, Leica, Germany). The sections were rinsed with phosphate‐buffered saline and incubated for 2 h in a blocking solution consisting of phosphate‐buffered saline supplemented with 5% bovine serum albumin and 0.3% Triton X‐100 (Acros Organics, Belgium). The sections were then incubated for 24 h with a primary antibody against tyrosine hydroxylase (rabbit polyclonal; 1:1000; catalog no. p21962, Invitrogen, USA) diluted in blocking solution. After removal of the primary antibody, the sections were thoroughly rinsed at least three times with phosphate‐buffered saline and incubated overnight at room temperature with Alexa Fluor 594‐conjugated donkey anti‐rabbit IgG (1:1000; Jackson ImmunoResearch, USA) diluted in phosphate‐buffered saline. Nuclear counterstaining was performed using 4’,6‐diamidino‐2‐phenylindole dihydrochloride, and the sections were mounted on slides using 10% glycerin. Images were acquired using a VS120 light microscope equipped with a 20× objective (Olympus) and an A1R confocal microscope (Nikon, Tokyo, Japan) equipped with a 40× objective.

### 2.7 Chemogenetic Manipulation

Before the experimental day, mice underwent a 5‐day handling period (30 min/day) and were acclimated to the head‐mounted EEG recording equipment for 30 min per day for 3 consecutive days in the testing environment. On the test day, animals were placed in the testing room 1 h before behavioral assessment to allow for acclimatization. Chemogenetic modulation of VTA GABAergic neurons was achieved by intraperitoneal administration of clozapine N‐oxide (CNO; 1 mg/kg). Thirty minutes after CNO administration, mice received dexmedetomidine (40 µg/kg) intraperitoneally. EEG recording was initiated immediately thereafter and continued for at least 60 min.

### 2.8 Statistical Analysis

Statistical analyses were performed using GraphPad Prism 7.0 (GraphPad Software, USA), MATLAB R2018b, and IBM SPSS Statistics 19 (IBM, USA). Data normality was assessed using quantile–quantile plots. Fiber photometry data were analyzed using either the Mann–Whitney *U* test or unpaired Student's *t*‐test, as appropriate. An unpaired Student's *t*‐test was used to compare the relative power of delta waves between groups. EEG data were analyzed using two‐way repeated‐measures analysis of variance (ANOVA), followed by Bonferroni post hoc tests. All statistical tests were two‐tailed, and *p* < 0.05 was considered statistically significant. Unless otherwise specified, data are presented as the mean ± standard error of the mean (SEM).

## 3 Results

### 3.1 Dexmedetomidine Inhibited VTA GABAergic Neurons

To investigate the effects of systemic dexmedetomidine administration on GABAergic neurons in the VTA, we injected a Cre‐dependent AAV‐EF1α‐DIO‐GCaMP6m (Broussard et al. 2018) into the VTA of Vgat‐Cre mice and used fiber photometry to monitor real‐time changes in intracellular Ca^2+^ signals (Figure 1A). Fluorescence imaging confirmed robust GCaMP6m expression, with minimal colocalization with tyrosine hydroxylase (TH) immunostaining (Figure 1B). Because the VTA comprises approximately 60%–70% dopaminergic neurons and 20%–30% GABAergic neurons, the observed expression pattern was consistent with preferential expression of GCaMP6m in VTA GABAergic neurons. Fiber photometry recordings showed that intraperitoneal saline administration did not significantly alter GCaMP6m fluorescence. In contrast, systemic dexmedetomidine administration induced a marked decrease in GCaMP6m fluorescence, which partially recovered as the sedative effect subsided. These findings demonstrate that dexmedetomidine suppresses the activity of VTA GABAergic neurons (Figure 1C). Quantitative analysis confirmed a significant difference between the saline and dexmedetomidine groups (Mann–Whitney *U* test; saline, 0.17 ± 0.31 vs. dexmedetomidine, –8.46 ± 1.07; mean ± SEM; *U* = 0, *p* = 0.008, *n* = 5; Figure 1D).

**FIGURE 1:**
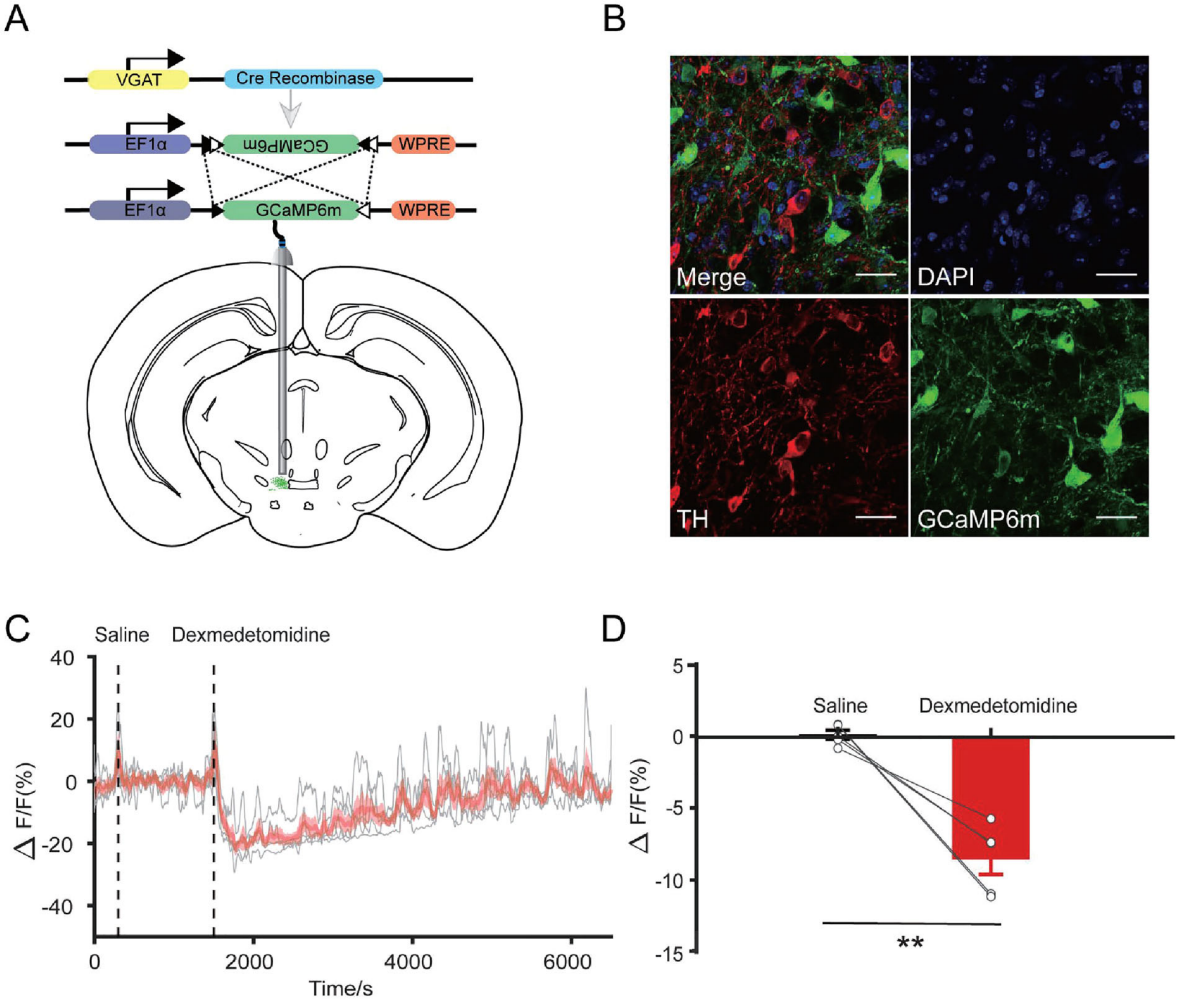
Systemic administration of dexmedetomidine inhibits GABAergic neurons in the ventral tegmental area. (A) Schematic diagram of fiber photometry recording. (B) Representative images showing tyrosine hydroxylase‐immunopositive neurons and GCaMP6m‐expressing neurons in the ventral tegmental area of a Vgat‐Cre mouse. Scale bar, 50 µm. (C) GCaMP6m fluorescence signals from VTA GABAergic neurons, time‐locked to the administration of saline or dexmedetomidine. Data from individual mice are shown in gray, and the mean signal across animals (*n* = 5) is shown in red, with red shading representing the SEM. (D) Quantification of GCaMP6m signals following saline and dexmedetomidine administration. Data are presented as mean ± SEM (Mann–Whitney *U* test; saline, 0.17 ± 0.31 vs. dexmedetomidine, −8.46 ± 1.07; *U* = 0, *p* = 0.008; *n* = 5). **p < 0.01.

### 3.2 Dexmedetomidine Increases GABAergic Transmission in VTA GABA Neurons

GABA is the principal inhibitory neurotransmitter in the central nervous system and plays a key role in regulating neuronal excitability. We therefore hypothesized that dexmedetomidine‐induced inhibition of VTA GABAergic neurons may be mediated by enhanced GABAergic input onto these neurons. To test this hypothesis, we injected a Cre‐dependent AAV‐DIO‐iGABASnFR (Marvin et al. 2018) into the VTA of Vgat‐Cre mice and used fiber photometry to monitor real‐time changes in GABA signaling in vivo (Figure 2A). Intraperitoneal saline administration resulted in minimal changes in iGABASnFR fluorescence (Figure 2B), with individual traces shown in Figure 2C. In contrast, dexmedetomidine administration produced a marked increase in GABA signals (Figure 2B), as further illustrated by the individual animal traces in Figure 2D. Quantitative analysis confirmed a significant difference between the dexmedetomidine and saline groups (unpaired *t*‐test; 95% confidence interval [CI], 11.49–20.97; saline, −1.83 ± 0.75 vs. dexmedetomidine, 14.40 ± 1.99; mean ± SEM; *t* = 7.63, *p* < 0.001; *n* = 6; Figure 2E).

**FIGURE 2:**
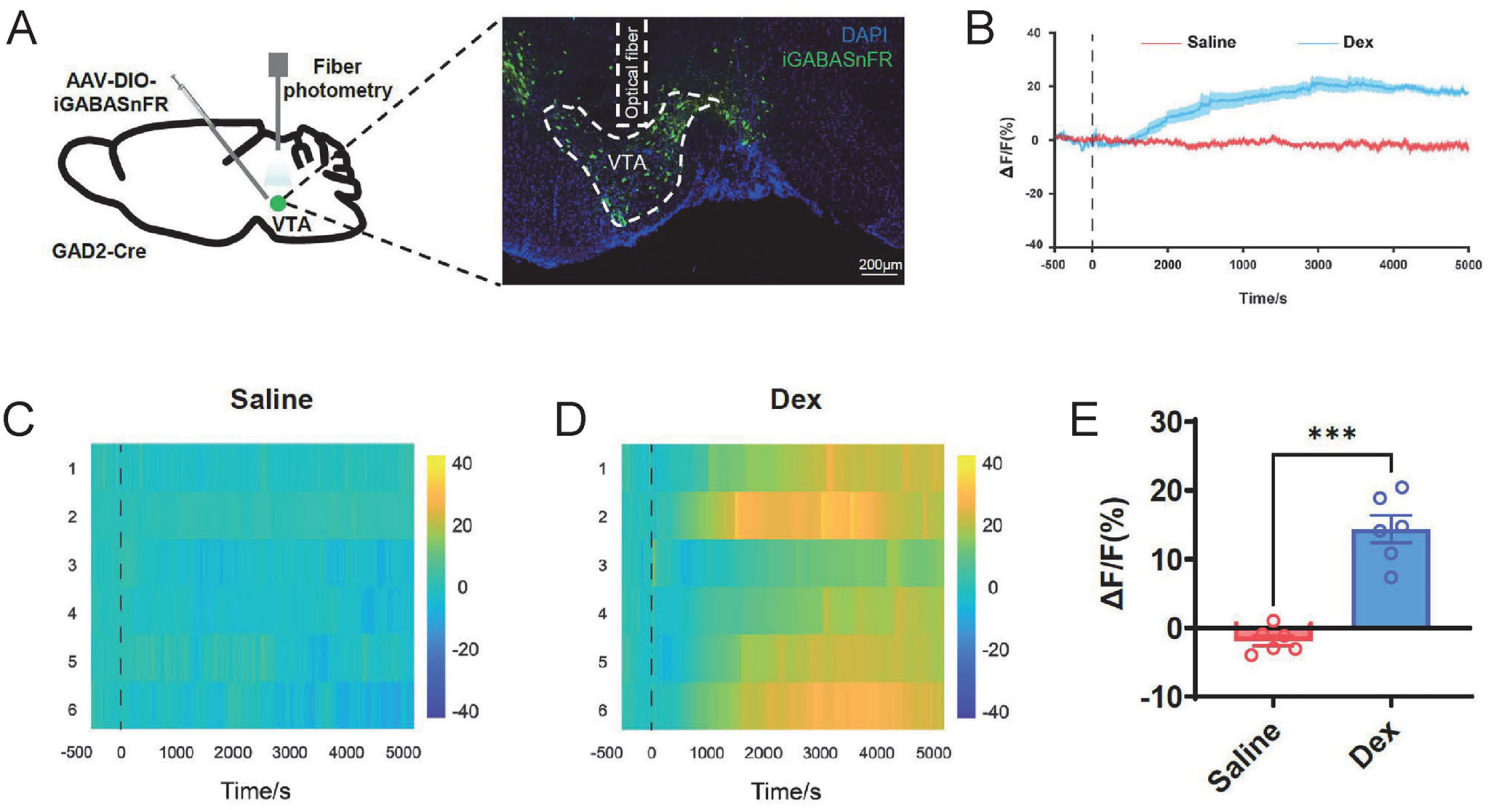
Dexmedetomidine increases GABAergic transmission in VTA GABAergic neurons. (A) Schematic diagram of fiber photometry recording (left) and fiber implantation above the VTA in GABAergic neurons expressing GCaMP6m (right). Scale bar, 200 µm. (B) iGABASnFR fluorescence signals from the VTA, time‐locked to the administration of saline or dexmedetomidine. (C) Heatmap showing changes in iGABASnFR fluorescence following intraperitoneal administration of saline, with individual traces shown. (D) Heatmap showing changes in iGABASnFR fluorescence following intraperitoneal administration of dexmedetomidine, with individual traces shown. (E) Quantification of iGABASnFR signals following saline and dexmedetomidine administration. Data are presented as mean ± SEM (unpaired *t*‐test; 95% CI, 11.49–20.97; saline, −1.83 ± 0.75 vs. dexmedetomidine, 14.40 ± 1.99; *t* = 7.63, *p* < 0.001; *n* = 6 mice). ***p < 0.001.

### 3.3 Chemogenetic Activation of VTA GABAergic Neurons Increases the Depth of Dexmedetomidine‐Induced Sedation

Because VTA GABAergic neurons have been implicated in sleep regulation, we investigated whether their activation affects the sedative effects of dexmedetomidine. Cre‐dependent AAV‐DIO‐hM3Dq‐mCherry or control AAV‐DIO‐mCherry was bilaterally injected into the VTA of Vgat‐Cre mice (Figure 3A). Three weeks later, all animals received CNO (1 mg/kg, intraperitoneally), followed 30 min later by dexmedetomidine (40 µg/kg, intraperitoneally), during which EEG recordings were performed. Compared with the mCherry control group, the hM3Dq group exhibited an apparent increase in low‐frequency EEG activity (Figure 3B). However, the reported between‐group difference in overall power density was not statistically significant (two‐way repeated‐measures ANOVA with Bonferroni correction; *F*(1, 22) = 0.25, *p* = 0.62; *n* = 12; Figure 3C). In contrast, delta‐band power differed significantly between groups (unpaired *t*‐test; 95% CI, −12.79 to −5.459; saline, 57.95 ± 1.51 vs. dexmedetomidine, 48.83 ± 0.92; mean ± SEM; *t* = 5.16, *p* < 0.0001; *n* = 12; Figure 3D).

**FIGURE 3:**
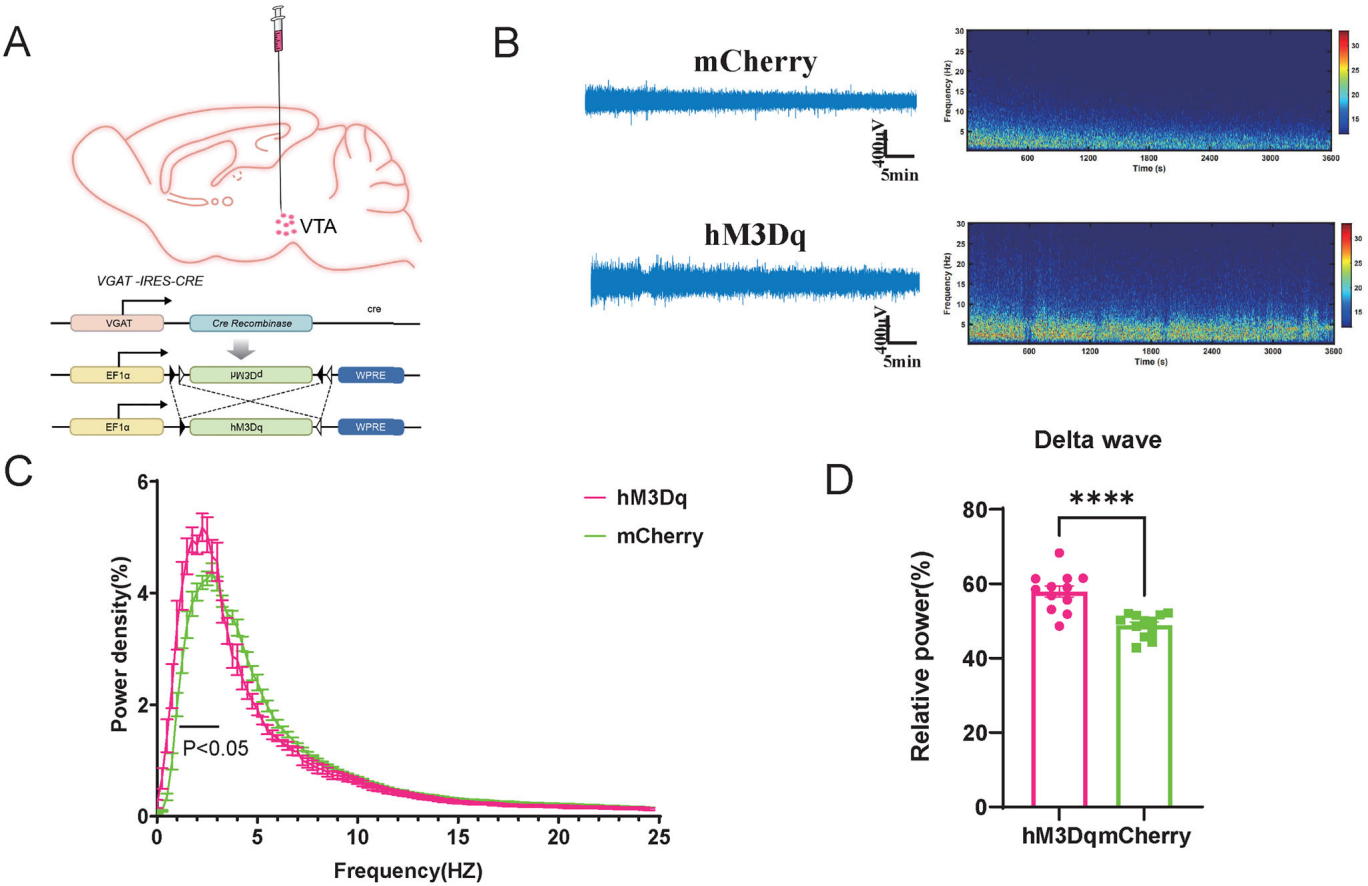
Chemogenetic activation of VTA GABAergic neurons increases the depth of dexmedetomidine‐induced sedation. (A) Schematic of stereotactic virus injection into the VTA of Vgat‐Cre mice. (B) Representative power spectral density plots of EEG recordings. Warm colors (e.g., red) indicate higher power at a given frequency, whereas cool colors (e.g., blue) indicate lower power. (C) EEG power density during dexmedetomidine‐induced sedation in the mCherry and hM3Dq groups (two‐way repeated‐measures ANOVA with Bonferroni multiple‐comparison test; *F*(1, 22) = 0.25, *p* = 0.62; *n* = 12). (D) Comparison of mean normalized relative cortical EEG delta power. Data are presented as mean ± SEM (unpaired *t*‐test; 95% CI, −12.79 to −5.459; saline, 57.95 ± 1.51 vs. dexmedetomidine, 48.83 ± 0.92; *t* = 5.16, *p* < 0.0001; *n* = 12). ****p < 0.0001.

### 3.4 Chemogenetic Inhibition of VTA GABAergic Neurons Attenuates the Depth of Dexmedetomidine‐Induced Sedation

To further investigate the role of VTA GABAergic neurons in dexmedetomidine‐induced sedation, we chemogenetically inhibited these neurons and assessed the effects on sedation. Cre‐dependent AAV‐DIO‐hM4Di‐mCherry was bilaterally injected into the VTA of Vgat‐Cre mice (Figure 4A). Three weeks later, all animals received CNO (1 mg/kg, intraperitoneally), followed 30 min later by dexmedetomidine (40 µg/kg, intraperitoneally), during which EEG recordings were performed. Compared with the control group, the hM4Di group exhibited reduced low‐frequency EEG activity (Figure 4B), with a significant between‐group difference in power density (two‐way repeated‐measures ANOVA with Bonferroni correction; *F*(1, 22) = 4.812, *p* = 0.04; *n* = 12; Figure 4C). Delta‐band power also differed significantly between groups (unpaired *t*‐test; 95% CI, 0.89 to 9.18; saline, 43.79 ± 1.78 vs. dexmedetomidine, 48.83 ± 0.92; mean ± SEM; *t* = 2.52, *p* = 0.02; *n* = 12; Figure 4D).

**FIGURE 4:**
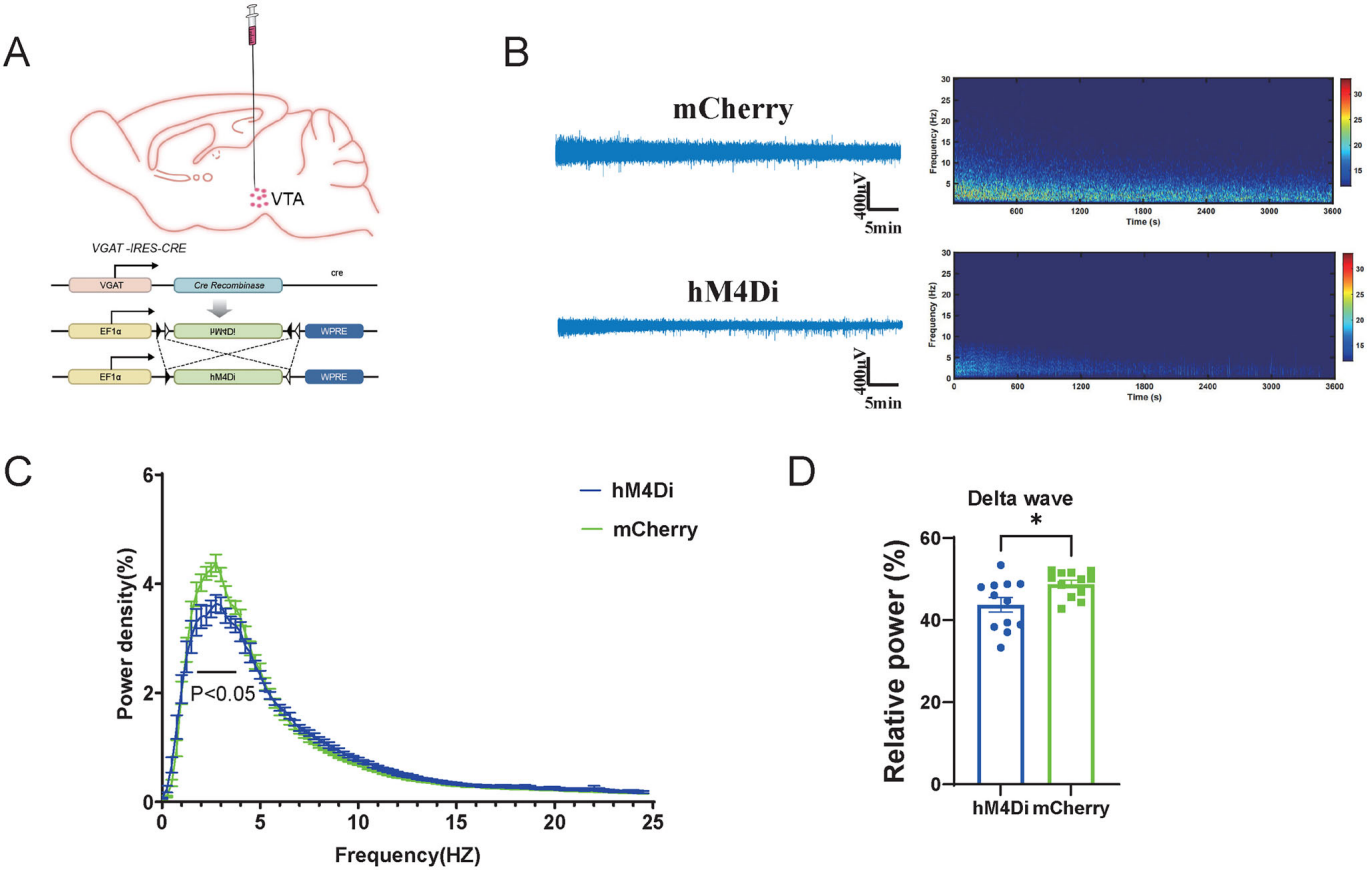
Chemogenetic inhibition of VTA GABAergic neurons attenuates the depth of dexmedetomidine‐induced sedation. (A) Schematic of stereotactic virus injection into the VTA of Vgat‐Cre mice. (B) Representative power spectral density plots of EEG recordings. Warm colors (e.g., red) indicate higher power at a given frequency, whereas cool colors (e.g., blue) indicate lower power. (C) EEG power density during dexmedetomidine‐induced sedation in the mCherry and hM4Di groups (two‐way repeated‐measures ANOVA with Bonferroni multiple‐comparison test; *F*(1, 22) = 4.812, *p* = 0.04; *n* = 12). (D) Comparison of mean normalized relative cortical EEG delta power. Data are presented as mean ± SEM (unpaired *t*‐test; 95% CI, 0.89 to 9.18; saline, 43.79 ± 1.78 vs. dexmedetomidine, 48.83 ± 0.92; *t* = 2.52, *p* = 0.02; *n* = 12). *p < 0.05.

## 4 Discussion

In this study, we combined EEG, in vivo calcium and GABA neurotransmitter imaging, and chemogenetics to investigate the mechanisms underlying dexmedetomidine‐induced arousable sedation. We found that dexmedetomidine suppresses VTA GABAergic neuron activity by enhancing GABAergic input to these neurons. Chemogenetic activation of VTA GABAergic neurons deepened sedation, whereas their inhibition attenuated it. These findings suggest that inhibition of VTA GABAergic neurons contributes to the arousable sedative effects of dexmedetomidine.

Our study demonstrated that dexmedetomidine increases GABAergic transmission in the VTA. This finding is consistent with the conventional view that anesthetic agents enhance central inhibitory signaling while reducing excitatory signaling. Previous studies have identified direct projections from POA GABAergic neurons to VTA GABAergic neurons and shown that activation of POA GABAergic neurons inhibits VTA GABAergic neurons (Gordon‐Fennell et al. 2020; Tao et al. 2023). Our previously published research also demonstrated that dexmedetomidine activates GABA neurons projecting from the POA to the VTA (GABA^POA‐VTA^ neurons). Thus, dexmedetomidine may activate GABA^POA‐VTA^ neurons, increasing GABA release in the VTA and subsequently inhibiting VTA GABAergic neurons. Our findings support this hypothesis.

VTA GABAergic neurons play an important role in sleep regulation. Yu et al. (2019) reported that activation of VTA GABAergic neurons promotes sleep, whereas their inhibition induces wakefulness in mice. Furthermore, Deng and colleagues demonstrated that activation of VTA GABAergic neurons facilitates isoflurane anesthesia (Yin et al. 2019). These findings suggest that VTA GABAergic neurons promote both sleep and anesthesia. Our finding that dexmedetomidine inhibits VTA GABAergic neurons therefore appears inconsistent with its sedative effects, which are widely attributed to inhibition of noradrenergic neurons in the LC. However, this apparent discrepancy may indicate that dexmedetomidine simultaneously engages both sedative and arousal‐promoting neural systems. The interaction between these systems may help maintain a relatively low arousal threshold and thereby contribute to arousable sedation. Consistent with this possibility, our chemogenetic experiments showed that activation of VTA GABAergic neurons deepened dexmedetomidine sedation, whereas their inhibition attenuated it.

An important question is how dexmedetomidine differs from commonly used procedural anesthetics, such as propofol, which are not typically associated with arousable sedation. Unlike dexmedetomidine, propofol potentiates GABA_A receptors, and previous studies have shown that propofol anesthesia suppresses VTA dopamine neuron activity (Zhu et al. 2023), consistent with its GABAergic mechanism of action. This difference in the effects of the two agents on VTA neural circuits may contribute to the distinct arousable sedative properties of dexmedetomidine.

Our study has several limitations. First, dexmedetomidine was administered intraperitoneally, whereas intravenous administration would more closely reflect its clinical use. From a pharmacokinetic perspective, intraperitoneally administered drugs are absorbed primarily through the portal circulation and undergo hepatic first‐pass metabolism, which can result in variable bioavailability and complicate precise pharmacokinetic characterization and we did not directly measure brain extracellular fluid concentrations, we cannot provide direct evidence that efficacious free levels were reached. Dexmedetomidine has also been reported to transiently inhibit gastrointestinal motility after intraperitoneal administration, potentially confounding studies involving gastrointestinal function. Although local tissue tolerance was acceptable in our experiment, intraperitoneal administration has been associated with mild tissue irritation and necrosis in some reports. Future studies should therefore consider intravenous administration to improve clinical relevance. Second, the upstream and downstream neural circuits through which VTA GABAergic neurons mediate the arousable sedation induced by dexmedetomidine require further investigation. Third, the proposed disinhibition of VTA dopamine neurons was not directly measured and therefore remains inferential. Future studies using direct measurements of dopaminergic activity are warranted to validate this proposed mechanism. Fourth, EEG was the sole measure of dexmedetomidine‐induced sedation depth in this study. Incorporating behavioral measures of arousal and sedation, such as responses to painful stimuli and changes in pupil size, would provide complementary evidence and strengthen the conclusions drawn from EEG findings. Fifth, the sample size was determined primarily on the basis of previous EEG studies investigating anesthetic mechanisms. Because no formal power analysis was performed, the adequacy of the sample size and the precision of the study estimates may be limited. Future studies should incorporate a priori power calculations to determine sample sizes appropriate for detecting clinically or biologically meaningful effects in mice. Sixth, although CNO was not a direct experimental variable in this study, its potential off‐target effects should be acknowledged. CNO is not entirely pharmacologically inert and may exert nonspecific effects through at least two mechanisms: (1) in vivo conversion to clozapine, which can interact with multiple endogenous monoaminergic receptors, and (2) direct interactions with endogenous G protein–coupled receptors or downstream signaling pathways independent of DREADDs. Although these potential effects are unlikely to substantially alter our conclusions, they cannot be completely excluded. Future studies should further evaluate the potential off‐target effects of CNO and, where feasible, incorporate appropriate control groups or alternative chemogenetic approaches to strengthen the mechanistic interpretation of the findings.

## 5 Conclusions

These findings suggest that dexmedetomidine enhances GABAergic input to VTA GABAergic neurons and inhibits their activity, potentially contributing to its arousable sedative effects. Further studies are needed to establish the causal role of this mechanism and to clarify the neural circuits involved.

## Author Contributions

**Jin Rao**: conceptualization, investigation, methodology, software, formal analysis, data curation, supervision, Writing – review and editing, validation, visualization, resources, Writing – original draft, project administration. **Gaolin Qiu**: funding acquisition, methodology, writing – review and editing. **Jiansheng Wang**: conceptualization, methodology, data curation, project administration, writing – review and editing. **Yuanhai Li**: conceptualization, writing – review and editing, visualization, validation, methodology.

## Funding

National Natural Science Foundation of China, Grant Number: 82101349

## Ethics Statement

All animal care and procedures strictly followed institutional policies and governmental regulations, and were approved by the Animal Ethics Committee of Anhui Medical University (Ethical Approval No. LLSC20210042).

## Conflicts of Interest

The authors declare no conflicts of interest.

## Data Availability

The data that support the findings of this study are available from the corresponding author upon reasonable request.
